# Supplementary material for: Machine Learning Framework for Characterizing Processing–Structure Relationship in Block Copolymer Thin Films
Source: Macromolecules. 2026 Jan 12;59(2):714–27. doi: 10.1021/acs.macromol.5c03272 (PMC12854768; doi:10.1021/acs.macromol.5c03272)
Supplement: Supplementary file 1 [file ma5c03272_si_001.pdf]

Supplementary Information: Machine Learning Framework for Characterizing Processing-Structure Relationship in Block Copolymer Thin Films

Bradley G. Lamb, Saroj Upreti, Yunfei Wang, Daniel Struble, Chenhui Zhu, Guillaume Freychet, Xiaodan Gu, Boran Ma

GISAXS bulk morphology analysis

The 2D GISAXS patterns were analyzed to verify the bulk thin film morphology was as expected. Previous analysis of the higher order peak spacing ratio was unable to determine the presence of hexagonally packed cylinders, due to the possible masking of the expected 11 reflection, resulting in the peak spacing ratios matching with both hexagonally packed cylinders and lamella. In the GISAXS 2D scattering pattern, the intensity distribution along the vertical (q_z) direction is not continuous or uniform; instead, it presents localized high-intensity regions (Fig. S1). This discrete modulation is characteristic of constructive interference from a hexagonally packed cylindrical morphology oriented parallel to the substrate.[1] This behavior can be observed further as the morphology transitions from vertically packed cylinders to horizontally packed cylinders (Fig. S1 a-c).

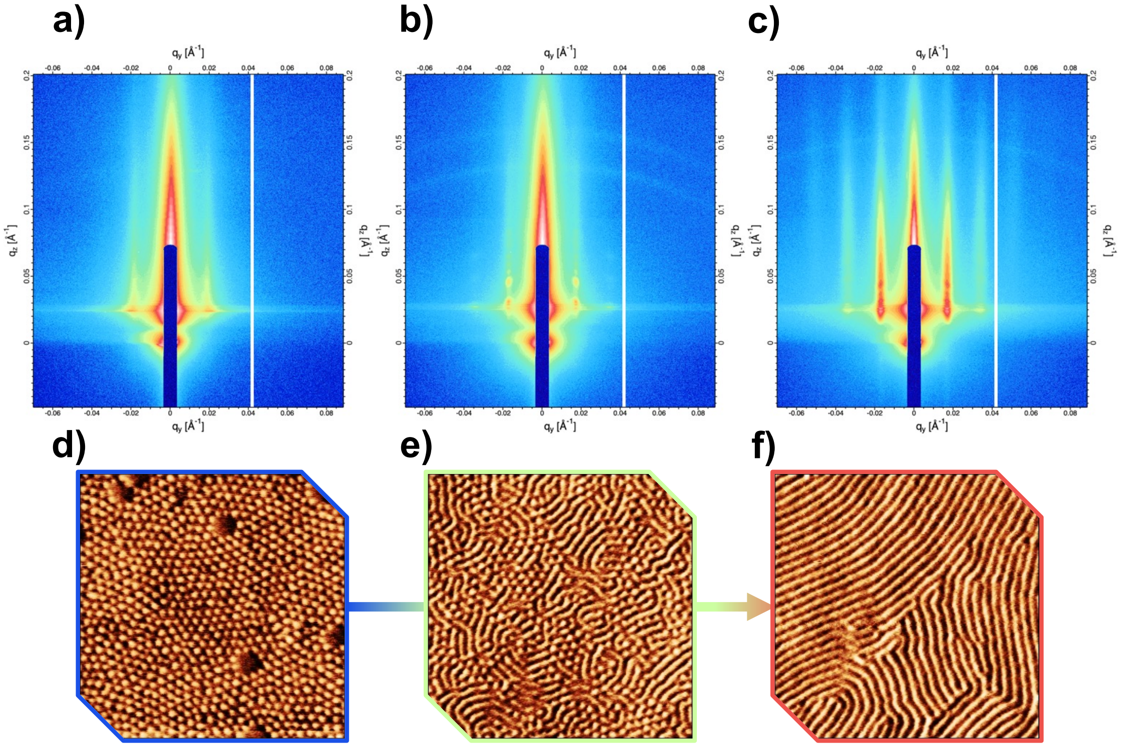

Figure S1. Block copolymer 2D GISAXS patterns processed with an 80% toluene:THF solvent ratio, chloronaphthalene additive at an additive ratio of a) 2, b) 4, c) 7. d-f) Representative AFM images, 1 μm × 1 μm, are shown to visualize the surface morphology at the processing conditions within each column.

Threshold determination for AFM grain size analysis

Prior to saving the final average grain size for each sample, a crude average was calculated using all available “grains” for each sample. This crude average, in some cases, contained small artificial grains that did not meet the criteria to be considered in larger neighboring grains. Therefore, a threshold, which was the product of the threshold parameter and the crude average, was applied to filter these artificial grains. The determination of the thresholding parameter was crucial to the accurate quantification of grain size. For example, in images with small, discrete grains maximizing the collection of grains is ideal, while still filtering small artificial grains. However, for images with larger grains, small artificial grains should be filtered out as to not artificially decrease the average grain size. A range of threshold parameters was tested, from 0% to 300%, corresponding to 0 and 3.0 (**Fig. S2**). A sharp increase in average grain size is observed at threshold value up to 1.0 (**Fig. S2 a**), resulting from the filtering of small artificial grains, circled in red (**Fig S2 b-d left**). Average grain size increases at a much lower rate between threshold values of 1.0 and 2.0, during which small artificial grains continue to be filtered out while maintaining true small grains, circled in green (**Fig. S2 b, c middle**). Upon increasing the threshold beyond 2.0, however, average grain size increases more rapidly as true small grains are filtered out, artificially increasing the average grain size, as seen by the lack of green highlighted grains (**Fig. S2 b, c right**). Therefore, a threshold of 1.5, 150%, was selected as it maintains true small grains, while reliably filtering small artificial grains.

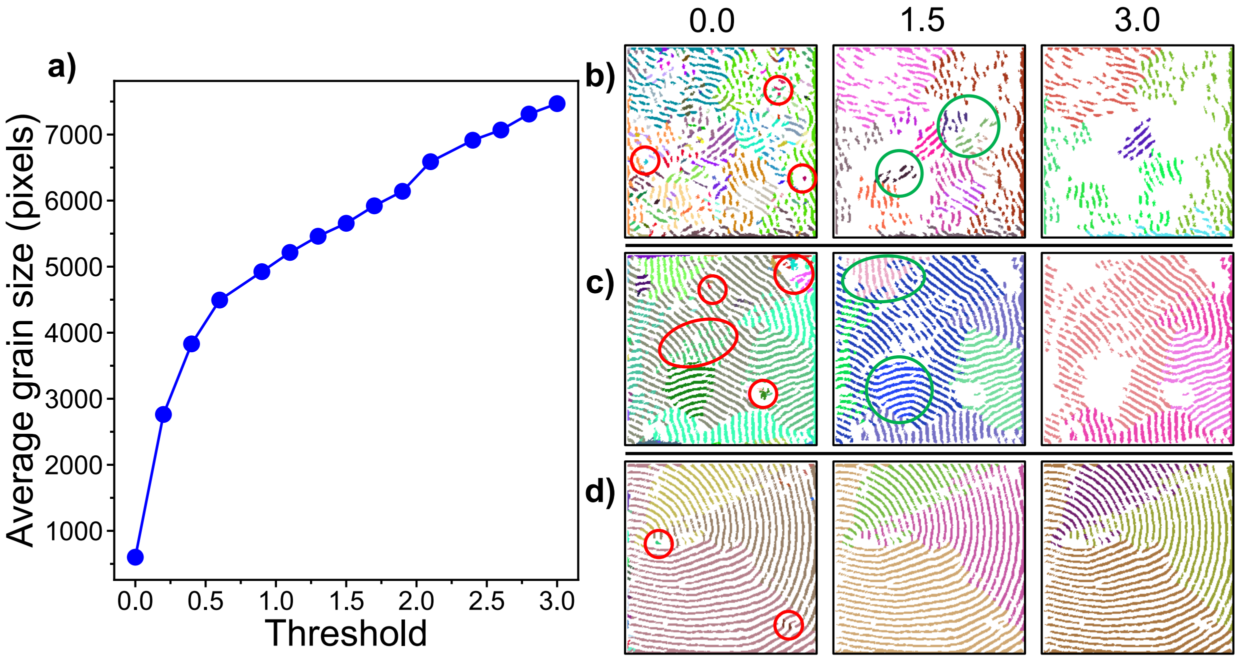

Figure S2. Effect of threshold value on average grain size a) revealing a sharp increase in grain size up to threshold value of 1.0, followed by marginal increases up to a value of 2.0 before increasing more significantly up to 3.0. AFM images with color-coded grains from threshold values ranging from 0.0 to 3.0 containing b) short-range grains, c) mid-range grains, and d) long-range grains with respect to each other.

Verification of AFM grain analysis code

The in-house grain analysis code presented in this work was tested on AFM images of BCP films from a similar system by Murphy et al. Their analysis process involved identifying the correlation length, or persistence length of parallel ordered domains. This value is often used interchangeably with grain size; however, it lacks 2D resolution which provides more robust measurements. Despite this, correlation length is still a valuable measurement and was used as a benchmark for the code presented in this study. The grains identified by the grain analysis code can be seen for each image (**Fig. S3 a-f**). Results are summarized in **Table S1**, along with results from Murphy et al for comparison. Except for image 5 (**Fig. S3 e**), which was hindered by

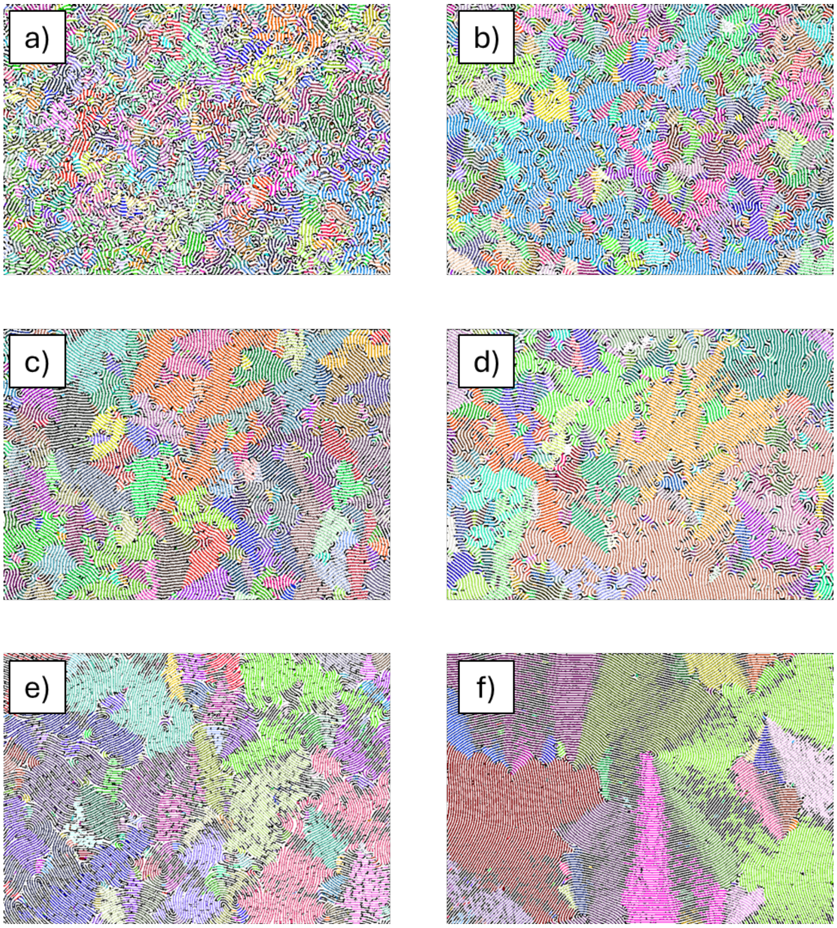

Figure S3. Block copolymer AFM images, 5 μm × 3.5 μm, a) 1, b) 2, c) 3, d) 4, e) 5, f) 6, from Murphy et al. with individual grains overlaid identified by the in-house grain analysis code.[2]

the quality of the image, results are comparable with that of Murphy et al. Despite the overall agreement in trends between our grain area measurements and the correlation length measurements reported by Murphy et al., the absolute magnitudes of the increases are not fully consistent.[2] In samples with smaller grains, the correlation length remains short and increases only gradually. Yet these grains can merge into long, continuous structures that do not exhibit extended 1D correlation lengths (**Fig. S3 b**). Our grain area measurements capture this behavior, showing large increases relative to the correlation length because they quantify the full

2D extent of the grains rather than a 1D projection. As the grains grow larger, however, the correlation length increases align more closely with the grain area increases, as seen in the transition from sample 4 to 6.

Table S1. Comparisons of correlation length from Murphy et al. and grain size from our grain analysis protocol.

	**1**	**2**	**3**	**4**	**5**	**6**
**Grain size (μm^2)**	0.0546	0.1560	0.2135	0.4300	0.3180	1.431
**Correlation length (nm)**	70	91	154	178	286	700

Convolutional Neural Network (CNN)

The framework for the CNN follows the modified VGG16 approach and is shown in detail below:

```
class DeeperCNN(nn.Module):
   def __init__(self, num_classes=3, num_filters1=32, num_filters2=64, num_filters3=128,
             kernel_size=5, dropout_rate=0.5):
      super(DeeperCNN, self).__init__()       # Block 1
      self.block1 = nn.Sequential(
         nn.Conv2d(1, num_filters1, kernel_size=kernel_size, padding=2),  # Padding set to 2 for
maintaining spatial size
         nn.BatchNorm2d(num_filters1),
         nn.ReLU(),
         nn.Conv2d(num_filters1, num_filters1, kernel_size=kernel_size, padding=2),
         nn.BatchNorm2d(num_filters1),
         nn.ReLU(),
         nn.MaxPool2d(kernel_size=2, stride=2)
      )       # Block 2
      self.block2 = nn.Sequential(
         nn.Conv2d(num_filters1, num_filters2, kernel_size=kernel_size, padding=2),
         nn.BatchNorm2d(num_filters2),
         nn.ReLU(),
         nn.Conv2d(num_filters2, num_filters2, kernel_size=kernel_size, padding=2),
         nn.BatchNorm2d(num_filters2),
         nn.ReLU(),
         nn.MaxPool2d(kernel_size=2, stride=2)
      )       # Block 3
      self.block3 = nn.Sequential(
         nn.Conv2d(num_filters2, num_filters3, kernel_size=kernel_size, padding=2),
         nn.BatchNorm2d(num_filters3),
         nn.ReLU(),
         nn.Conv2d(num_filters3, num_filters3, kernel_size=kernel_size, padding=2),
         nn.BatchNorm2d(num_filters3),
```

```
        nn.ReLU(),
        nn.MaxPool2d(kernel_size=2, stride=2)
    )        # Fully connected layers
    self.flatten = nn.Flatten()
    self.fc_layers = nn.Sequential(
        nn.Linear(512 * 16 * 16, 512),  # Corrected size
        nn.ReLU(),
        nn.Dropout(p=dropout_rate),
        nn.Linear(512, 128),
        nn.ReLU(),
        nn.Dropout(p=dropout_rate),
        nn.Linear(128, num_classes)
    )    def forward(self, x):
    x = self.block1(x)
    x = self.block2(x)
    x = self.block3(x)
    x = self.flatten(x)
    x = self.fc_layers(x)
    return x
```

Morphological data from GISAXS and AFM

Scatter plots of the GISAXS- and AFM-measured data are shown below (**Fig. S4**) with respect to additive ratio and solvent ratio. Clear trends can be seen for each property, which change as a function of the additive ratio. However, these relationships are much clearer for the GISAXS-measured data, whereas the AFM-measured data is much more variable, with data spread across a large range.

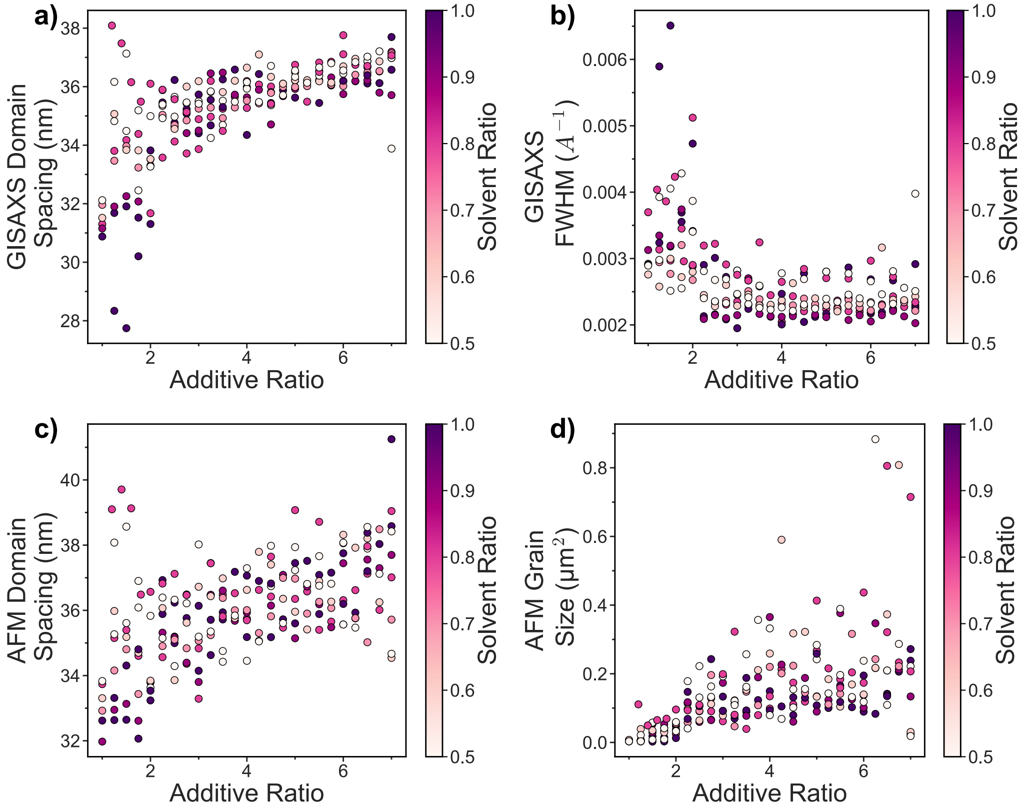

Figure S4. Scatter plots of a, b) domain spacing and FWHM measured by GISAXS, and c, d) domain spacing and grain size measured by AFM respectively. Each property is plotted as a function of the additive ratio, with a color bar related to the solvent ratio

ML Regression Hyperparameter search space

Model performance was evaluated using random forest, linear, lasso, and Bayesian ridge regression (**Fig. S5**). The random forest model showed consistently higher testing R^2 values for GISAXS-measured domain spacing and FWHM, as well as AFM-measured domain spacing. In contrast, predictions of AFM-measured grain size remained weaker across all methods. That outcome tracks with the local-variability inherent to AFM measurements, which raises the effective noise floor and limits any model's ability to capture reliable trends. For the GISAXS-based properties, the random forest's advantage reflects the stronger signal quality of the measurements and the presence of nonlinear processing-property relationships that the linear models cannot resolve. As a result, the random forest captures a larger fraction of the underlying variance than the more restrictive linear and regularized approaches.

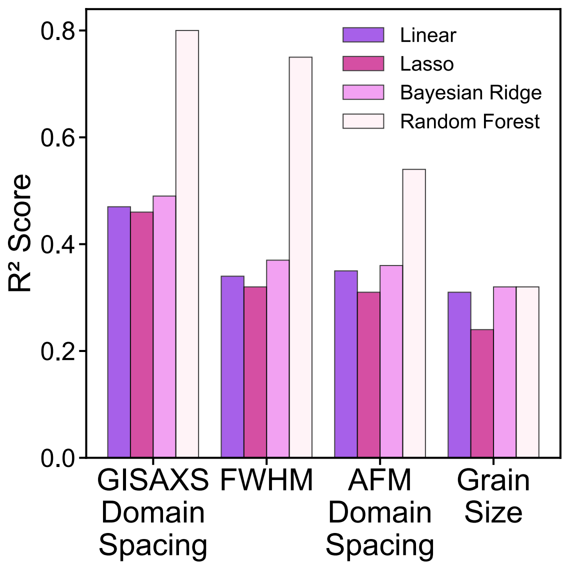

Figure S5. Comparison of R^2 across the targeted outputs for random forest, linear, lasso, and Bayesian ridge regression.

Random forest:

```
param_grid = {
    'n_estimators': [10, 25, 50, 75, 100],
    'criterion': ["squared_error", "friedman_mse"],
    'max_depth': [None, 5, 10, 20, 30],
    'min_samples_split': [2, 5, 10, 20],
    'max_leaf_nodes': [None, 10, 20, 30, 50],
    'max_features': ['sqrt', 'log2', 1.0],  # 1.0 = use all features
    'bootstrap': [True, False]
}
```

Multi-layer perceptron:

```
param_grid = {
    'mlp__hidden_layer_sizes': [
        (10,), (20,), (30,), (50,),
        (30, 10), (50, 20), (50, 25, 10), (100,), (100, 50)
    ],
```

```
    'mlp__activation': ['relu', 'tanh'],
    'mlp__solver': ['adam'],
    'mlp__alpha': [1e-5, 1e-4, 1e-3, 1e-2],
    'mlp__learning_rate': ['constant', 'adaptive'],
    'mlp__learning_rate_init': [0.001, 0.01, 0.05],
    'mlp__beta_1': [0.9, 0.95],  # momentum-like parameter for adam
    'mlp__beta_2': [0.999, 0.99],  # controls stability/speed tradeoff
}
```

Support vector machine:

```
param_grid = {
    'kernel': ['rbf', 'linear', 'poly', 'sigmoid'],
    'C': [0.01, 0.1, 1, 10, 100],
    'epsilon': [0.001, 0.01, 0.1, 0.2, 0.5],
    'gamma': ['scale', 'auto', 0.001, 0.01, 0.1, 1],
    'degree': [2, 3, 4],  # only used for 'poly' kernel, but no harm including it
    'coef0': [0.0, 0.5, 1.0]  # used in 'poly' and 'sigmoid'
}
```

XGBoost:

```
param_grid = {
    'learning_rate': [0.01, 0.05, 0.1],
    'max_depth': [3, 5, 7],
    'subsample': [0.7, 0.8, 1.0],
    'colsample_bytree': [0.7, 0.9, 1.0],
    'reg_lambda': [1, 5, 10],
    'reg_alpha': [0, 0.5, 1],
    'n_estimators': [100, 150, 200],
    'objective': ['reg:squarederror'],
    'eval_metric': ['rmse']
}
```

Effect of training data size on machine learning performance

The effect of training set size was assessed by fitting random forest models with optimized hyperparameters to progressively smaller subsets of the GISAXS data. These models were chosen for comparison due to their strong performance relative to those trained on AFM-based properties. The testing R^2 for both domain spacing and FWHM drops sharply at a training size of ~115 (**Fig. S6**). For domain spacing, R^2 continues to decline steadily as the training set shrinks, while for FWHM it remains relatively stable until decreasing sharply at ~50 samples.

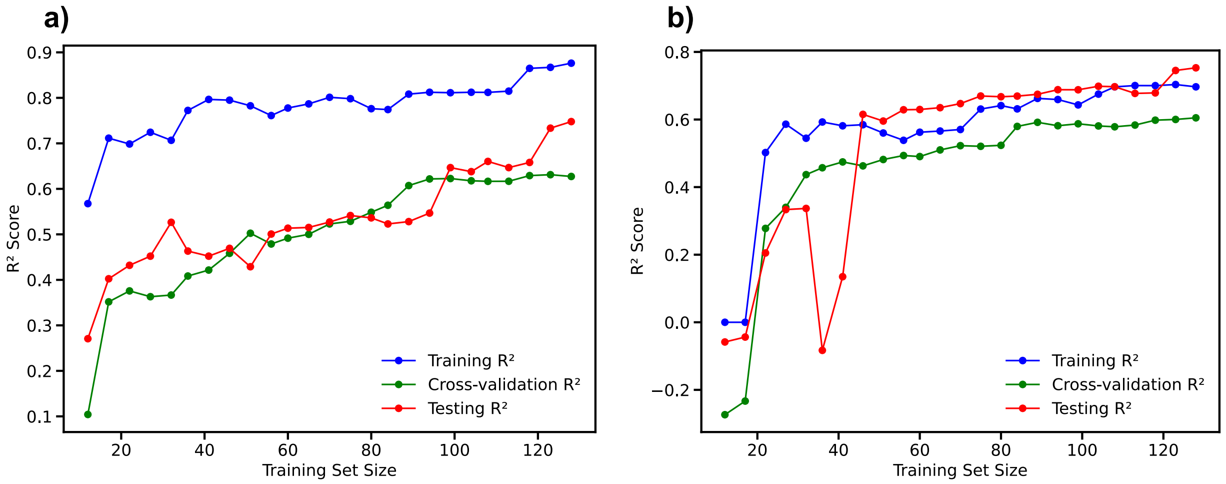

Figure S6. Training curves showing R^2 for training, cross-validation, and testing sets as a function of dataset size for random forest models trained on GISAXS a) domain spacing and b) FWHM data.

Morphological data predictions by machine learning

Parity plots and error distributions from each of the four machine learning models, RF, SV, XGB, and MLP are shown in the figures below for each of the four morphological properties characterized in this study (**Figs. S7-10**). Performances varied significantly across the different models when predicting the GISAXS-measured data, domain spacing and FWHM. Interestingly, the tree-based models, RF and XGB, performed well when predicting the GISAXS data when compared to MLP and SV, which is likely a result of the small dataset size being mapped effectively by a series of trees opposed to more complicated methods of MLP which would likely perform better with a larger dataset to learn from. Despite this, all of the models demonstrated similar, poor performances when predicting the AFM-measured data. This is likely the results of the highly variable, local measurements from AFM being difficult to account for by the models. Therefore, independent of model architecture, the AFM-measured data was unable to be effectively mapped.

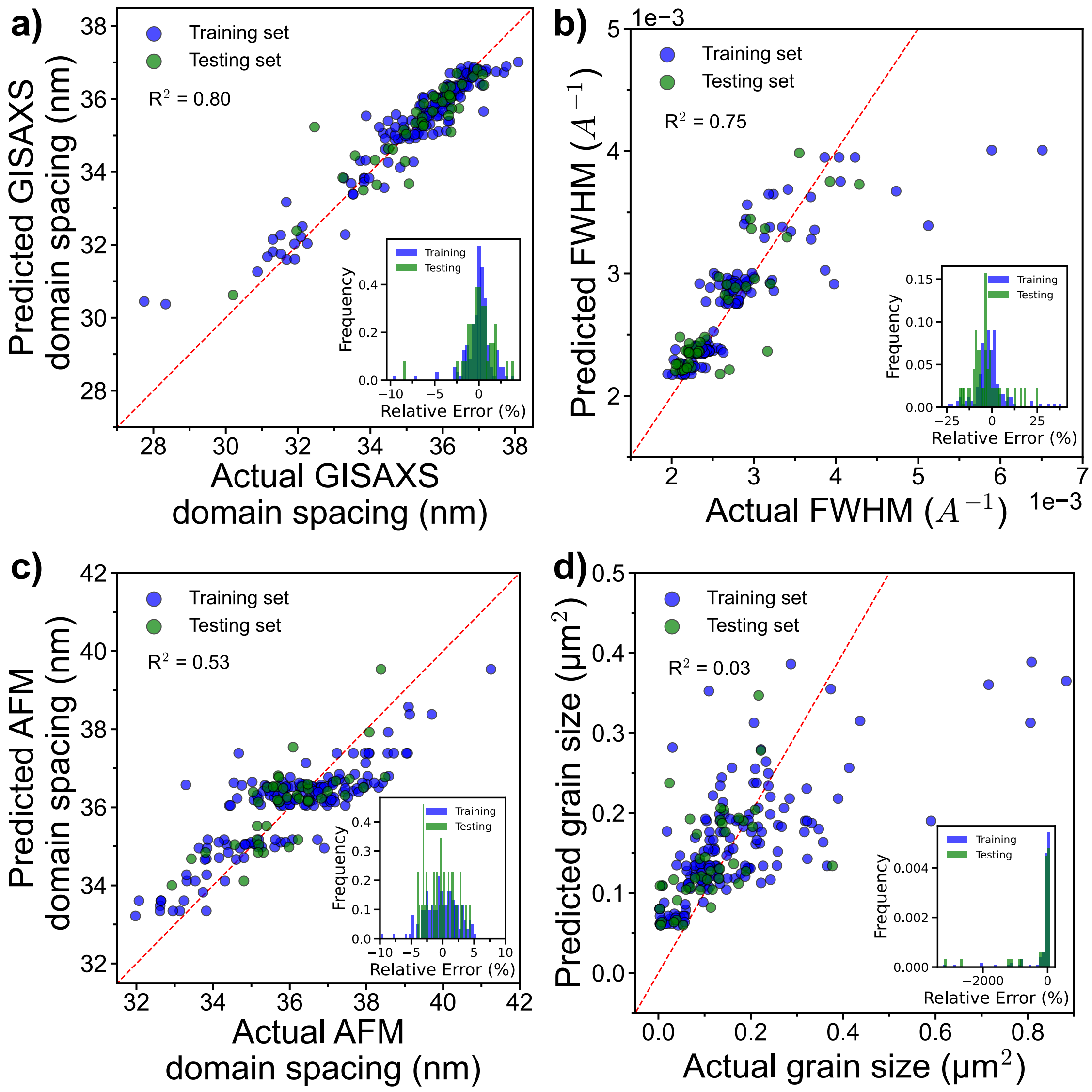

Figure S7. Parity plots and error distribution insets from the random forest regression models trained on a, b) domain spacing and FWHM measured by GISAXS, and c, d) domain spacing and grain size measured by AFM respectively.

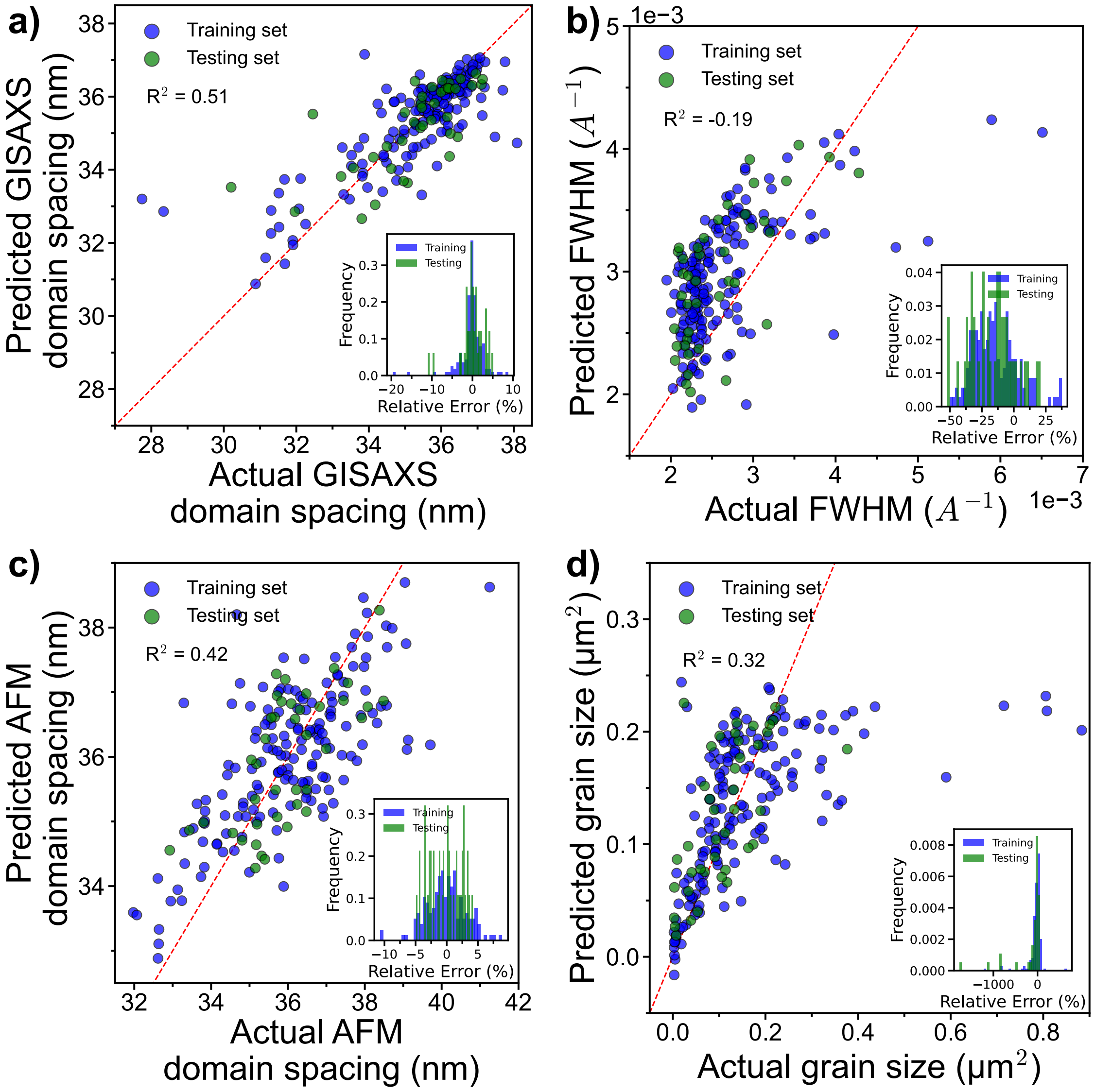

Figure S8. Parity plots and error distribution insets from the support vector regression models trained on a, b) domain spacing and FWHM measured by GISAXS, and c, d) domain spacing and grain size measured by AFM respectively.

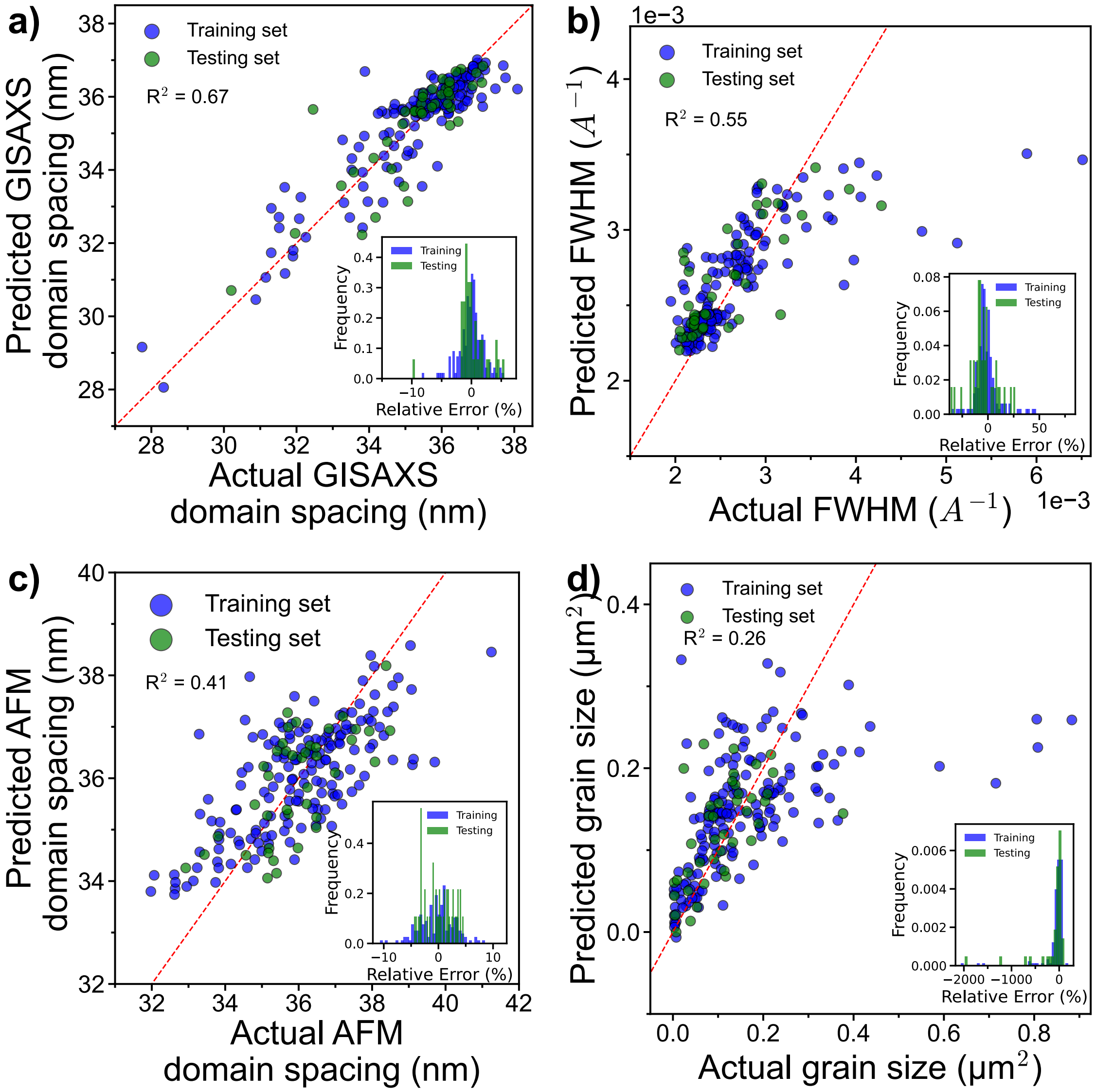

Figure S9. Parity plots and error distribution insets from the multi-layer perceptron regression models trained on a, b) domain spacing and FWHM measured by GISAXS, and c, d) domain spacing and grain size measured by AFM respectively.

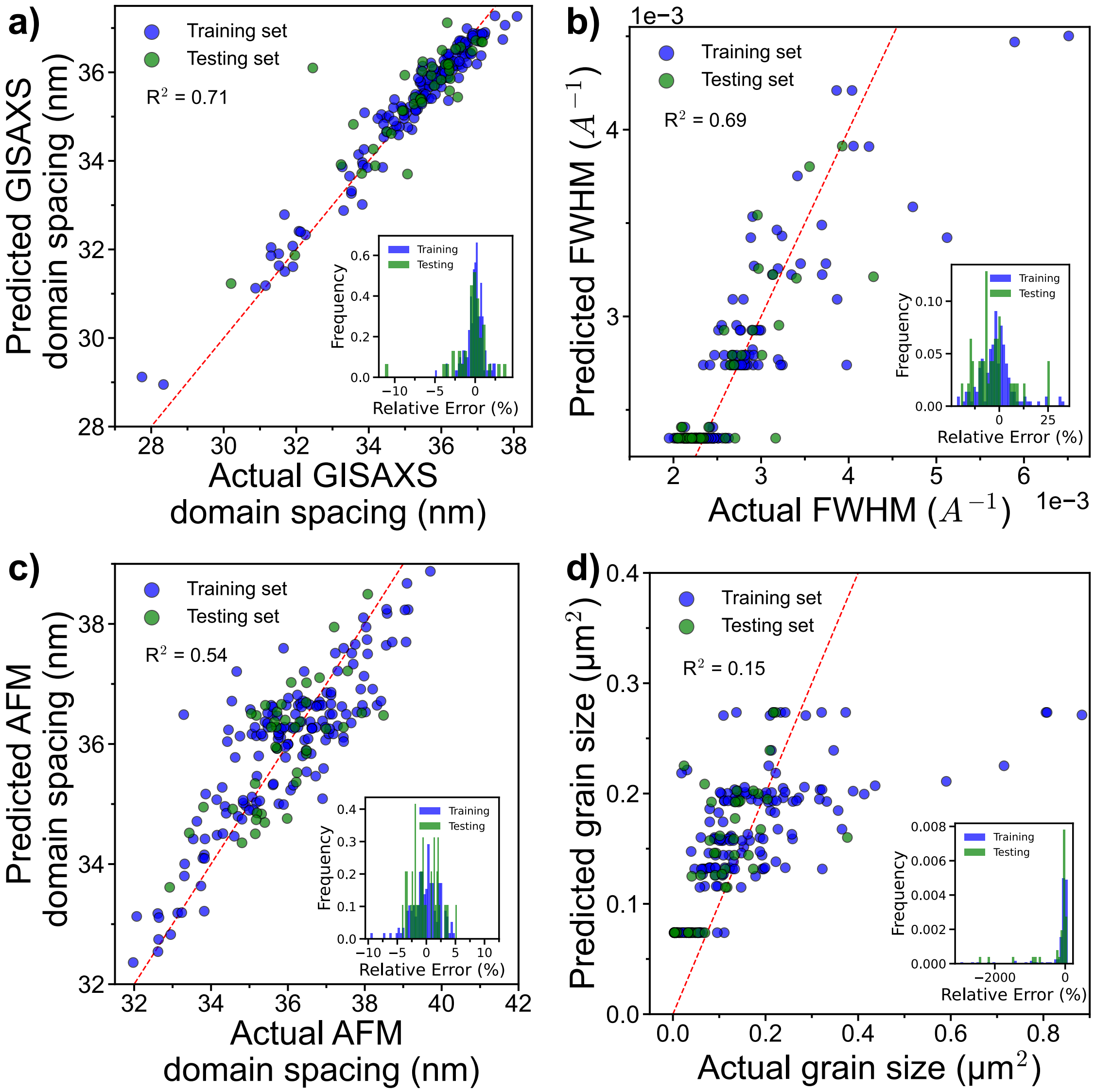

Figure S10. Parity plots and error distribution insets from the XGBoost regression models trained on a, b) domain spacing and FWHM measured by GISAXS, and c, d) domain spacing and grain size measured by AFM respectively.

SHAP interaction plots

SHAP interaction plots are shown below for the models trained on the GISAXS-measured domain spacing and FWHM (**Figs. S11, S12**). All six combinations of the processing parameters have been plotted with respect to each other to visualize the dependance they have on one another. For both the models trained on domain spacing and FWHM, the most apparent relationship appears to be between the additive ratio and solvent ratio, which is discussed in the main text.

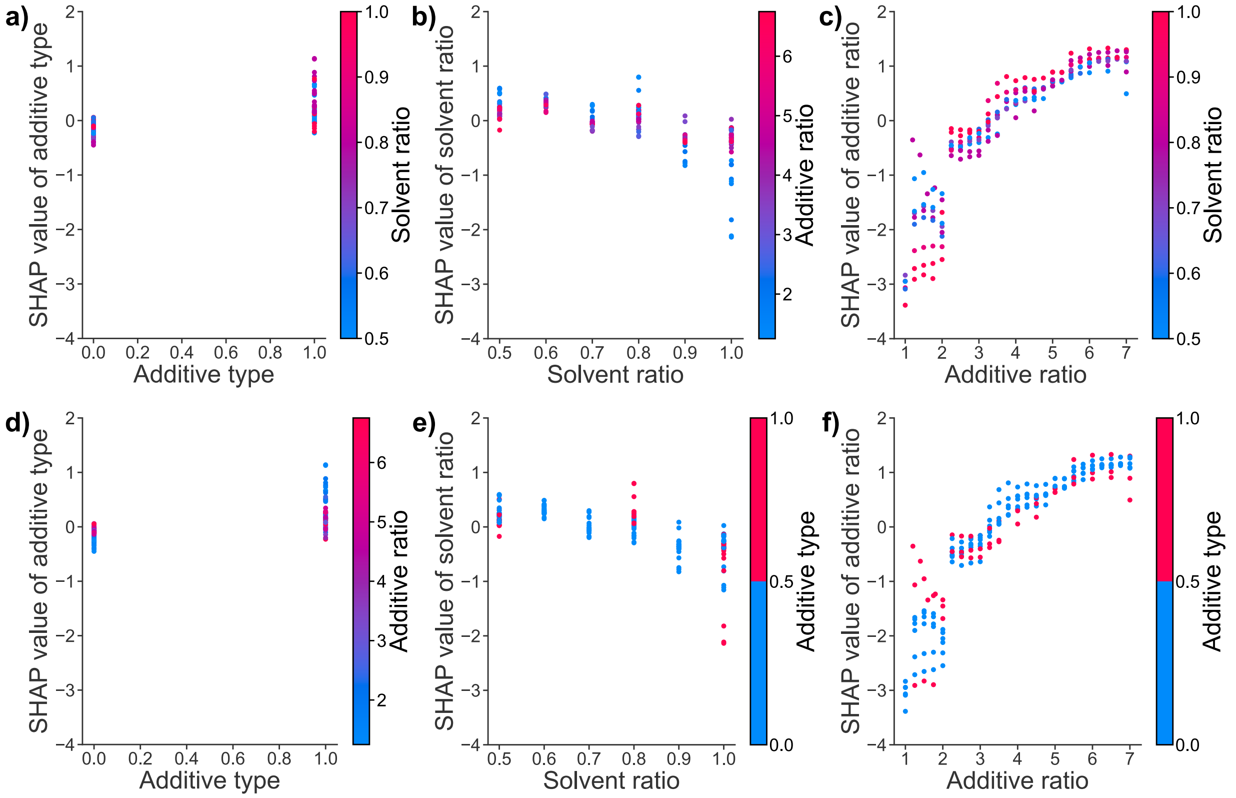

Figure S11. SHAP interaction plots from the model trained on GISAXS-measured domain spacing for a) additive type and solvent ratio, b) solvent ratio and additive ratio, c) additive ratio and solvent ratio, d) additive type and additive ratio, e) solvent ratio and additive type, and f) additive ratio and additive type.

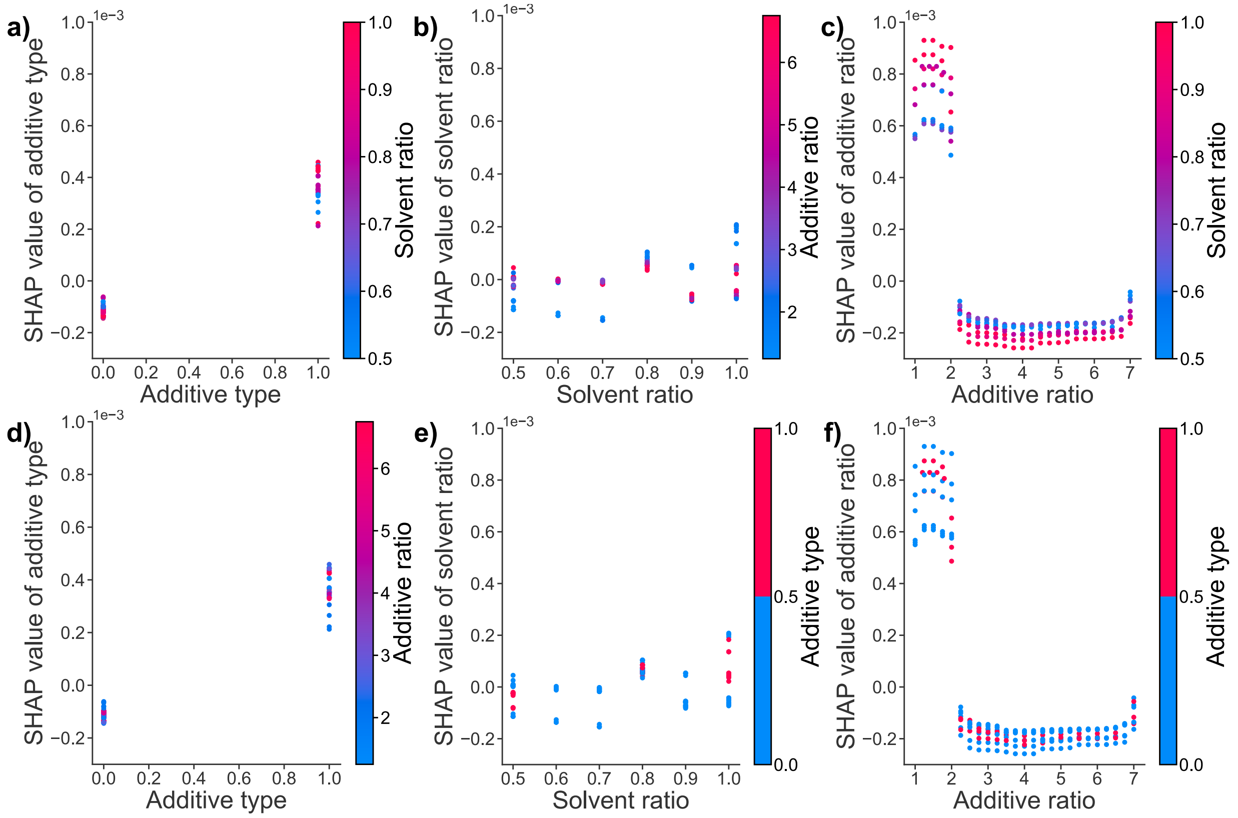

Figure S12. SHAP interaction plots from the model trained on GISAXS-measured FWHM for a) additive type and solvent ratio, b) solvent ratio and additive ratio, c) additive ratio and solvent ratio, d) additive type and additive ratio, e) solvent ratio and additive type, and f) additive ratio and additive type.

References

(1) Gu, X.; Gunkel, I.; Hexemer, A.; Gu, W.; Russell, T. P. An In Situ Grazing Incidence X-Ray Scattering Study of Block Copolymer Thin Films During Solvent Vapor Annealing. Advanced Materials 2014, 26 (2), 273–281. https://doi.org/10.1002/adma.201302562.

(2) Murphy, J. N.; Harris, K. D.; Buriak, J. M. Automated Defect and Correlation Length Analysis of Block Copolymer Thin Film Nanopatterns. PLOS ONE 2015, 10 (7), e0133088. https://doi.org/10.1371/journal.pone.0133088.
